# Antimicrobial and Antioxidant Activity of Apricot (*Mimusopsis comersonii*) Phenolic-Rich Extract and Its Application as an Edible Coating for Fresh-Cut Vegetable Preservation

**DOI:** 10.1155/2022/8440304

**Published:** 2022-10-21

**Authors:** Emília Maria França Lima, Caroline Harumi Silva Matsumura, Geovana Luísa da Silva, Isabela Cristina Soares Patrocínio, Catarina Angeli Santos, Patrícia Aparecida Pimenta Pereira, Neuza Mariko Aymoto Hassimotto, Uelinton Manoel Pinto, Luciana Rodrigues da Cunha

**Affiliations:** ^1^Food Research Center, Department of Food and Experimental Nutrition, Faculty of Pharmaceutical Sciences, University of Sao Paulo, Sao Paulo SP, Brazil 05508-000; ^2^Department of Foods, Federal University of Ouro Preto, Ouro Preto, Minas Gerais, Brazil 35400-000

## Abstract

Edible coatings have several advantages in preserving foods, such as avoiding water loss, controlling microbial growth, and reducing the need for preservatives added directly to the product. Antimicrobial action can be obtained by adding antimicrobial substances including phenolic compounds commonly found in plant extracts. This study evaluated the phenolic compounds content, antioxidant and antimicrobial activity of pulp, and seed extracts of *Mimusopsis comersonii* (popularly known in Brazil as *abrico*), besides the phenolic compounds were identified and quantified in the pulp extract. Edible coatings were incorporated with pulp extract in order to evaluate the preservation of minimally processed apples and *baroa* potatoes against foodborne bacteria, and enzymatic browning was also determined. Myricetin-3-glucoside, quercetin-3-glucoside, and kaempferol-3-glucoside were identified as major flavonoids in the apricot pulp extract. The seed and pulp extracts inhibited all tested microorganisms, especially *Staphylococcus aureus* and *Salmonella* Typhimurium. Edible coatings added with 9% of phenolic extract showed *in vitro* antimicrobial activity, in addition to being effective in preventing enzymatic browning in minimally processed apples and *baroa* potatoes for up to 15 days of storage. They were also effective in reducing up to 2 log CFU/g of aerobic mesophiles after 15 days of storage for apples, even though no microbial inhibition in *baroa* potatoes was observed under the same conditions. The addition of pulp phenolic extract in edible coatings proved to be an alternative in the preservation of apples and in the antibrowning activity of minimally processed *baroa* potatoes.

## 1. Introduction

The demand for fresh-cut or minimally processed vegetables (MPVs), or minimally processed products, grows continuously due to the practicality and health benefits of these foods. The shelf-life of MPVs is reduced when compared with their intact counterparts, since fresh-cut involves the disruption of plant tissues [1, 2]. Minimal processing induces respiration and the production of ethylene, affecting enzymatic browning, degradation of the lipids in the cellular membrane, and the production of secondary compounds and water loss [3]. Among these consequences, the enzymatic browning limits the shelf-life of these products, and different antibrowning methods have been investigated to prolong their durability [2, 4–6].

Food packaging is mostly made of plastics due to low price, durability, and excellent barrier properties of these materials. In the recent past, the packing market has shown a rate of growth of 3.5% per year and an estimate of US $ 997 billion in 2020. The food sector is responsible for almost half of the packaging market consumption [7, 8].

The concern with this volume of nonbiodegradable plastics discarded in the environment has intensified efforts for creating more renewable natural biopolymers. Pectin is polysaccharide which is part of the plant cell wall [9, 10]. It is amorphous, nontoxic, and soluble in water and has outstanding ability to form films and the potential applicability in active food packaging [10, 11]. Edible pectin films and their derivatives are mainly used in the packaging of fruits and vegetables. In addition, recent studies show that the incorporation of natural ingredients, including essential oils, phenolic compounds, and fruit extracts, enhances the antioxidant, antimicrobial, and mechanical properties of filmogenic solutions [10, 12–17].

Beach-apricot fruit (*Mimusopsis comersonii*), also known as tree passion fruit and yellow apricot, belongs to the *Sapotaceae* family, and it is originally from Madagascar. In Brazil, it is known as *abrico*, and it is considered an exotic fruit, but widespread in the coastal region due to its tolerance to sandy and saline soils [18]. It has globose berries, hard skin, yellow color, and spherical or flat shape. Its pulp is floury and yellowish and may contain up to two dark seeds. It can be eaten fresh, even though it is not well appreciated, or used in confectionery to prepare sweets, creams, liqueurs, and ice cream [19]. The processing of apricot and fruits in general results in byproducts or waste material such as kernel and seeds, containing valuable biological compounds, such oils, dietary protein, and fiber [20]. In this context, apricot kernel oil can be used in many applications as in the food industry, cosmetics, and in medicinal supplements [20, 21].

This study evaluated the content of phenolic compounds, antioxidant, and antimicrobial activity of *M. comersonii* extracts (pulp and seed) and their incorporation into edible coatings for preservation of MPVs, focusing on antibrowning treatment.

## 2. Materials and Methods

### 2.1. Plant Material Preparation

Beach-apricot (*abricó-da-praia*) (*Mimusopsis comersonii*) fruits were harvested during the fruiting season, between December and March of 2017, in the mature stage, in Viçosa, Minas Gerais, Brazil (coordinates 20° 45′ 17^″^ S, 42° 52′ 57^″^ W). The fruits were rinsed in water and immersed in a solution of 50 mg/L of sodium hypochlorite solution for 15 min and then washed again with water to remove residual chlorine [22]. Seed were manually detached from the pulp, and both were kept at -80°C for further use.

### 2.2. Pulp and Seed Extracts of *M. comersonii*

#### 2.2.1. Preparation of Pulp and Seed Crude Extracts

Apricot extracts were obtained as described by Bertoldi et al. [23], with modifications detailed by Santos et al. [16], and the extracts were kept at -80°C.

#### 2.2.2. Content of Total Phenolic Compounds in Extracts

The content of total phenolic compounds in the pulp and the seed extracts was evaluated by the Folin-Ciocalteau assay, using a standard curve of gallic acid according to Waterhouse [24]. The results were expressed as mg GAE/g of pulp or seed as detailed by Santos et al. [16].

#### 2.2.3. Characterization of Phenolic Compounds in Pulp Extract

The identification and quantification of the phenolic content of *M. comersonii* pulp extract were performed as previously described by our group Quecán et al. [25], following the protocol detailed in Fraga et al. [26]. The phenolic standards used in the current study were quercetin 3-glucoside, myricetin-3-glucoside, kaempferol-3-glucoside (Extrasynthese, Genay, France) monitored at 370 nm, and gallic acid (Sigma-Aldrich) monitored at 270 nm.

#### 2.2.4. Antioxidant Activity

The determination of the antioxidant activity was performed by using DPPH (2,2-diphenyl-1-picrylhydrazyl), according to Brand-Williams et al. [27], and ABTS (2,2′-Azino-bis(3-ethyl-benzothiazoline-6-sulfonic acid) radicals, according to Re et al. [28]. The analyses were carried out protected from light, covering all glassware with aluminum foil. The results were expressed as EC_50_ (gram of fresh mass per gram of DPPH) which is the concentration of extract in which 50% of the radical is reduced. For the ABTS assay, trolox-6-hydroxy-2,5,7,8-tetramethylchroman-2-carboxylic acid was used as reference substance. The results were expressed as micromoles of Trolox equivalents (TEs) per gram of fresh weight (*μ*mol of TEs/g f.w.).

#### 2.2.5. Antimicrobial Action of Extracts

The antimicrobial effect of the extracts was determined by the minimum inhibitory concentration (MIC) and inhibitory potential (IP) assays, as previously described by our research group [16]. The tests were evaluated against a panel of microorganisms including the proteobacteria *Escherichia coli* ATCC 10536, *Salmonella enterica* serovar Typhimurium ATCC 14028, *Pseudomonas aeruginosa* PA14, and gram-positive strains of *Listeria monocytogenes* ATCC 7644 and *Staphylococcus aureus* ATCC 6538P.

The MIC was performed following the method described by Wiegand et al. [29] with modifications adopted by Santos et al. [16]. The concentrations of pulp extract ranged from 1.1 to 8.8 mg GAE/g, and the seed extract ranged from 0.41 to 3.3 mg GAE/g of phenolic extract in each microtube. The MIC was considered as “the lowest concentration of extract in which there was no bacterial growth observed” [16].

For IP determination, the microtubes were prepared as described previously for MIC. Then, the samples were serially diluted and spread plated on LB agar. Plates were incubated for 24 h, and colonies were counted [16]. The results were expressed as the inhibitory potential (IP), calculated according to the following equation.

(1) $$\begin{array}{c} \text{IP}=\log\left(\frac{No}{N}\right). \end{array}$$

In which *N*_0_ denotes the count in CFU/mL of the control (LB without extract); *N* denotes the count in CFU/mL of the treatment in the evaluated concentration. An IP value of 1 means that the treatment had 10 times less cells than the control [16].

### 2.3. Edible Coatings Incorporated with Phenolic-Rich Extracts of *M. comersonii*

#### 2.3.1. Production of Edible Coatings

Edible coatings were prepared according to Ayala et al. [30] and Melgarejo-Flores et al. [31]. The filmogenic mixture was made with 1 g of pectin (Rica Nata®), 10 mL of sterile distilled water, and 0.5 mL of glycerin, stirring and heating until complete homogenization. Subsequently, 1 mL of pulp or seed extracts was incorporated into the filmogenic solution, homogenized, and deposited in sterile Petri dishes and incubated at 25°C/6 h. After drying, the coatings were cut into discs (*d* = 2 cm) for further testing. The control coatings were also prepared without the addition of extracts.

#### 2.3.2. Antibacterial Activity of Edible Coating

The antimicrobial activity was determined by inhibitory potential (IP) in LB broth against *E. coli* ATCC 25922, *L. monocytogenes* ATCC 35152, *S.* Typhimurium, *S. aureus* ATCC 22923, and *P. aeruginosa* PA14, as described previously. The test evaluated discs with 2 cm diameter of edible coatings with 9% (*v*/*v*) of pulp and seed phenolic extracts.

#### 2.3.3. Application of Edible Coatings in a Food Matrix

Edible coatings incorporated with pulp extract of *M. comersonii* were applied on apples (*Malus domestica* Borkn) and *baroa* potatoes (*Arracacia xanthorrhiza*) obtained at a local grocery store. After selection, they were rinsed and decontaminated in sodium hypochlorite solution at 200 mg/L for 15 minutes, as recommended for MPVs [32]. The vegetables were peeled and cut manually into cubes of approximately 1 cm^2^ with stainless steel knives, previously cleaned.

Twenty cubes of vegetables were immersed for 2 minutes in 24 mL antioxidant solution (0.1% N-acetylcysteine (*w*/*v*), 1% ascorbic acid (*w*/*v*), 2% citric acid (*w*/*v*), and 1% (*w*/*v*) calcium chloride) to avoid enzymatic browning of the food matrices during the experiment and, subsequently, drained for 30 seconds. Then, the cubes were immersed in the incorporated coatings with the pulp extract (9% *v*/*v*) or control without extract, for 3 minutes. After draining, the cubes were placed in polypropylene pots and kept for 15 days, under refrigeration. Color analysis and counting of aerobic mesophilic microorganisms were performed to evaluate the shelf life at times 0, 3, 6, 8, 12, and 15 days.

#### 2.3.4. *In Situ* Instrumental Color Analysis

The color of vegetables cubes was determined in triplicate using a digital colorimeter Chroma Meter CR-400 (Konica Minolta, Japan) to detect the parameters: L^∗^ (brightness), a^∗^ (red/green coordinate), and b^∗^ (yellow/blue coordinate). From the values obtained from these coordinates, we calculated the browning index (BI) using the following equations [33].

(2) $$\begin{array}{c} \text{BI}=\frac{100\;\left(X-0.31\right)}{0.172},\\ x=\frac{\left(a^{*}+1.75L^{*}\right)}{5.645L^{*}+a^{*}-3.02b^{*}}. \end{array}$$

#### 2.3.5. *In Situ* Antimicrobial Activity

The antimicrobial activity of edible coatings was assessed by counting aerobic mesophiles. At predetermined times, the cubes of vegetables of each treatment were weighed (1 g) and diluted in 9 mL of 0.1% (*w*/*v*) peptone water and homogenized in Stomacher (Marconi, Brazil) for 1 minute. Then, the samples were diluted and spread plated on PCA (Plate Count Agar). The samples were incubated at 32° C/24 h, the colonies were counted, and the results were expressed in log CFU/g. All tests were executed in triplicate at times 0, 3, 6, 9, 12, and 15 days.

### 2.4. Statistical Analysis

Experiments were done with three replicates and evaluated by analysis of variance (ANOVA) following Tukey's test (*p* < 0.05) in Statistical Analysis System and Genetics Software [34].

## 3. Results and Discussion

### 3.1. Phenolic Compounds in *M. comersonii* Extracts

The content of phenolic compounds present in the pulp of *M. comersonii* was 17.66 ± 0.52 52 mg GAE/g of pulp. The seed extracts contained 6.59 ± 0.52 mg GAE/g of seed. These results indicate that the pulp has a higher amount of total phenolic compounds than the seed (*p* < 0.05). There are no reports on bioactive compounds of *M. comersonii* in the literature, so we compared the data with different species of apricot. Rao et al. [35] analyzed the *Mimusops elengi* extract and obtained a higher value of phenolic compounds than that found in the present study (698.7 mg GAE/g of pulp). A lower value (0.25 mg GAE/g of pulp) was observed for apricots from Para (*Mammea Americana*) by Braga et al. [36]. Fruits can be classified as having low (<1 mg GAE/g of sample), medium (1-5 mg GAE/g of sample), and high (>5 mg GAE/g of sample) content of phenolic compounds [37]. Therefore, pulp and seed of *M. comersonii* could be categorized as possessing a high content of phenolic compounds. These compounds have the ability to donate hydrogen or electrons and also prevent oxidation of food ingredients such as lipids [38].

**Figure 1:**
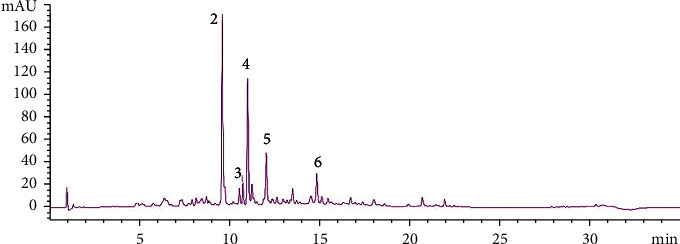
Chromatogram at 370 nm of pulp phenolic extract of *M. comersonii* attained by high-performance liquid chromatography with diode array detector (HPLC-DAD). Peaks identified peak 2: myricetin-3-glucoside; peak 3: quercetin rutinoside; peak 4: quercetin-3-glucoside; peak 5: kaempferol-3-glucoside; peak 6: myricetin glycoside. (Peak 1 of 3-O-galloyl quinic acid/gallic acid was obtained at 270 nm; data not shown.)

Figure 1 shows the chromatogram of pulp phenolic extract of *M. comersonii.* The peaks' identification is shown in Table 1. Supplementary Figure S1 shows results of LC-qTOF-MS/MS that detail the mass spectrum obtained in the study.

Two phenolic acids presenting molecular ions at m/z 343 and at m/z 169 were identified as 3-O-galloyl quinic acid and gallic acid, respectively (Table 1). The flavonols myricetin-3-glucoside (m/z 479), quercetin-3-glucoside (m/z 463), and kaempferol-3-glucoside (m/z 447) were the major flavonoids identified in apricot pulp extract (Figure 1, peaks 2, 4, and 5).

Myricetin has important bioactive activity, displaying multiple preclinical biological effects, such as antimicrobial, antioxidant, neuroprotective, anticancer, immunomodulatory, and cardioprotective activities [39]. Myricetin showed a significant inhibitory action against *Salmonella* Cholerasuis, *Salmonella* Enteritidis, and *E. coli* [40]. The treatment with this compound causes morphological changes in bacteria, by altering the structure of the cell wall with consequent outflow of cytoplasmic content, as observed in *E. coli* O157:H7 and *S.* Cholerasuis [40]. Conversely, the use of myricetin as a food preservation agent is linked to its property acting as a protecting agent against lipid oxidation [41].

The polyphenols quercetin and kaempferol are one of the most common compounds found in fruits and vegetables and also have antimicrobial and quorum quenching action [42, 43]. For instance, Quecán et al. [25] observed that quercetin aglycone and quercetin 3-*β*-D-glucoside inhibited the motility of *P. aeruginosa* PAO1 and *Serratia marcescens* MG1, besides reducing the violacein production by *Chromobacterium violaceum*, a quorum sensing model organism. Adamczak et al. [44] investigated the inhibition of *S. aureus* PriA (SaPriA) helicase, which is crucial for bacterial survival. They showed that kaempferol binds to DNA helicase and hinders its ATPase activity. They further concluded that flavonoids may be used in the development of antibiotics.

### 3.2. Antioxidant and Antimicrobial Activity of *M. comersonii* Phenolic-Rich Extracts

The antioxidant activity of *M. comersonii* extracts is presented in Table 2. When using the DPPH radical, we observed a greater antioxidant activity of the pulp than the seed extract. However, using the ABTS radical, the seed showed greater activity. According to Hassimoto et al. [45] and Souza et al. [46], different results may be obtained when working with methods to determine the antioxidant activity, and these issues arise because of changes in the biological material as well as the choice of methods.

Both pulp and seed extracts had a MIC higher than the tested concentrations, which were 8.8 and 3.3 mg GAE/g of fresh sample, respectively, for all evaluated bacteria (*E. coli*, *L. monocytogenes*, *P. aeruginosa* PA14, *S.* Typhimurium, and *S. aureus*).

On the other hand, all the tested microorganisms had their populations reduced by the addition of extracts (Table 3). Greater IP of pulp was observed for *E. coli* and *S. aureus*, with more than six log reduction in their population, compared to the control. *S. aureus* was also strongly inhibited by the seed extract, with its population reduced by seven log cycles. Lower inhibitory effect of the pulp was observed for *L. monocytogenes* (Table 3).

No studies were found on the IP of pulp and seed extract of *M. comersonii*, which highlights the importance of this work. However, the antimicrobial activity of other fruit extracts is widely reported, such as the inhibition of *E. coli* (5.8 logs) and *S. aureus* (6.5 logs) by *grumixama* (*Eugenia brasiliensis*) extracts, as reported by Rodrigues et al., [47]; the inhibition of *E. coli*, *P. aeruginosa*, *L. monocytogenes*, and *S. aureus* by jambolan (*S. cumini* (L.) Skeels) extracts up to 8 logs reported by Santos et al. [16]; and the inhibition of *S. aureus* by *pitanga* (*Eugenia uniflora*) phenolic extract reported by Carvalho et al. [14], demonstrating that phenolic-rich extracts have considerable antimicrobial activity. The potential of phenolic compounds to inhibit bacterial growth and cell-to-cell communication in foodborne bacteria has also pointed to encouraging applications in the pharmaceutical and food fields [48]. These results indicate the potential use of fruit extracts as natural antimicrobials, as an application in edible coatings for food preservation.

### 3.3. Antibacterial Activity of Edible Coatings with Phenolic-Rich Extracts

The results of the IP of edible coatings incorporated with 9% of pulp and seed extracts of *M. comersonii* against *E. coli*, *S.* Typhimurium, *L. monocytogenes*, *S. aureus*, and *P. aeruginosa* are shown in Table 4. All bacteria showed sensitivity to the coatings incorporated with both phenolic extracts, promoting a reduction of at least 2 logs in the population of these microorganisms.

The antimicrobial activity may be related to the high content of phenolic compounds present in the pulp extract (17.66 mg AGE/g of pulp) and apricot seed extract (6.59 mg AGE/g of seed). Phenolic compounds act by destabilizing and increasing the permeability of the bacterial cell membrane and also by promoting changes in enzymes essential for bacterial cell metabolism [49].

Our study corroborates the findings by other authors. For instance, Santos et al. [16] observed antimicrobial activity against foodborne bacteria by cellulose acetate films incorporated with jambolan phenolic extract. Likewise, Carvalho et al. [14] observed the reduction of 4 log cycles in *S. aureus* population by cellulosic films incorporated with crude extract of *grumixama*. Dannenberg [50] evaluated cellulose acetate coatings added with the essential oil of pink pepper and found a reduction of up to 3 logs in the population of *S. aureus* and *L. monocytogenes*.

For the next tests, it was decided to use coatings incorporated with phenolic pulp extract due to the higher yield and content of phenolic compounds (17.66 ± 0.52 mg GAE/g of pulp) and greater antioxidant activity (EC50 of 478.62 ± 7.14 g sample/g DPPH) in relation to the seed extract.

### 3.4. Evaluation of Edible Coatings with Phenolic-Rich Pulp Extracts *In Situ*

Apple (*Malus domestica* Borkn) is a perishable fruit, and the main cause of deterioration in fresh-cut is the rapid action of enzymatic browning [51]. *Baroa* potato (*Arracacia xanthorrhiza*) usually undergoes rapid spoilage, mainly due to deterioration caused by microorganisms [52]. Both vegetables are extensively consumed in Brazil.  Table 5 shows the browning index (BI) of minimally processed apples and *baroa* potatoes with edible coatings added or not with phenolic-rich extracts of *M. comersonii* pulp.

Apples treated with *M. comersonii* pulp extract (AE) had lower browning rates (*p* ≤ 0.05) than the control treatment (AC) after 12 days of storage under refrigeration, showing the effectiveness of the *M. comersonii* pulp extract to prevent browning in this fruit. It is also observed that in 6-, 9-, and 12-days apples treated with extract (AE) showed a tendency of stability in relation to browning. *Baroa* potatoes darken over time for both treatments. However, the browning was significantly lower for the treatment with *M. comersonii* pulp extract (PE) at all times (*p* ≤ 0.05), showing a protective effect of phenolic-rich extracts of *M. comersonii* pulp in the potato browning.

This browning protective action for both foods can be explained by the presence of phenolic compounds and the consequent antioxidant activity of such molecules. Substances with antioxidant activity have protective capacity acting in several stages of the oxidative process, delaying damage due to oxidation by inactivating free radicals, which are unstable [14]. These compounds work by giving hydrogen or electron atoms, converting the radicals into stable substances, thus reducing the damage due to oxidation [38].

The enzymatic browning occurs by the reaction of polyphenol oxidase (PPO) enzyme with phenolic compounds in the presence of oxygen, resulting in the dark brown pigment formation [1, 3]. Because fresh-cut apple slices have a limited shelf life due to enzymatic browning, different antibrowning methods have been tested. Chung and Moon [3] observed that the composition of gases in the storage affects the browning index of sliced apples. Remorini et al. [4] observed that fresh-cut apples had a significantly reduced browning when treated with chlorine dioxide (100 mg/L) and ascorbic acid (3%). Martínez-Hernández et al. [53] observed that the addition of *α* and *β* cyclodextrins to apple juice obtained by high pressure processing reduced browning. Finally, Zha et al. [2] showed that riboflavin inhibits the browning of fresh-cut apples during 8 days under refrigeration, by inhibiting polyphenol oxidase and peroxidase. In the present study, we evaluated a longer storage time (15 days) aiming at an extension of shelf life of fresh-cut apple.

Nascimento and Canteri [5] evaluated the antibrowning effect of sodium metabisulfite (SMB) and ascorbic acid (AA) in ready-to-eat processed potatoes. The processing induced browning reactions, and the authors concluded that only SMB (0.5%) and combination of SMB (0.25%) and AA (0.25%) controlled darkening. In addition, Meng et al. [6] demonstrated that harvest influences browning by inducing the increase of tyrosine in late harvested potatoes. This indicates the importance of choosing an ideal harvest maturity for processing.

The microbiological quality of food has great importance because in addition to sensory and nutritional changes, and it can also affect consumers' health. The fresh-cut vegetables are more prone to the multiplication of microorganisms, as the tissues are more exposed to the high availability of moisture and nutrients compared to the intact vegetable [54]. The counting of aerobic mesophiles has been one of the most commonly used microbiological indicators of food quality, and this determination also allows obtaining information on the probable shelf life of the product [55].  Table 6 shows the counts of aerobic mesophiles in minimally processed apples and *baroa* potatoes with edible coatings added or not of phenolic-rich extracts of *M. comersonii* pulp.

For apples, microbial growth over time was observed for both treatments, with no significant difference between them (*p* > 0.05) until the 9th day of storage under refrigeration. From the 12th day on, apples coated with pulp extract (AE) had a significantly lower aerobic mesophilic count (*p* ≤ 0.05) than the control treatment (AC). The reduction of microbial growth in approximately 2 logs at the end of storage shows the effectiveness of the phenolic pulp extract in increasing the shelf life of apples in microbiological terms.

For *baroa* potatoes, there was no significant difference (*p* > 0.05) between aerobic mesophilic counts for treatment (PE) and control (PC) over storage, showing that phenolic-rich extract was not effective at inhibiting the growth of these bacteria in *baroa* potatoes.

Natural extracts and phenolic compounds have been used in edible coatings to reduce the growth of microorganisms, while maintaining the quality of food products [15]. However, these compounds may not behave effectively in all foods due to the influence of specific characteristics of the food, such as composition, acidity, light exposure, and storage temperature [14, 56]. Additionally, the inhibitory activity of antimicrobial agents can be influenced by the components of the food matrix, since the bioactive compounds can interact with food components, making them less available to act on microorganisms [57]. In addition, natural extracts have many bioactive compounds with different cellular targets and mechanisms [16].

Ramos-Garcia et al. [58] have shown the effect of chitosan coatings with natural compounds such as beeswax and lime essential oil to control microorganisms in fresh tomatoes stored at two temperatures. Likewise, Ventura-Aguilar et al. [59] showed that cinnamon essential oil and *Roselle calyces* extract added to chitosan edible films can be an effective at inhibiting the fungal *Colletotrichum fragariae* attack on strawberries. Hernandez-López et al. [60] developed a chitosan film with *α*-pinene and showed that it suppressed the growth of *Alternaria alternate* and maintained the physicochemical quality of bell pepper during cold storage. Carvalho et al. [14] demonstrated the effect of cellulosic film incorporated with grumixama extract against *Staphylococcus aureus.* Wong et al. [56] studied the use of *Centella asiatica* extract in the preservation of MPVs at room temperature and chilled storage. They observed different profiles of microbial counts according to fresh-cut vegetable (potatoes, lettuce, pineapple, and apple) and storage conditions. They concluded that the effect of the extract on the microbiological quality and color of foods was dependent on temperature and storage period, in addition to the characteristics of the fruits and vegetables.

Other alternatives for reducing the growth of microorganisms and extending freshness of fresh-cut vegetables are modified atmosphere packaging for minimally processed apples [61], combined use of nisin and citric acid for sliced onion [62] and the use of ultrasound for disinfecting fresh-cut lettuce [63].

## 4. Conclusions

The crude extracts of the pulp and seed of *M. comersonii* showed *in vitro* antimicrobial activity, being effective in controlling several bacteria of relevance in food microbiology. The edible coatings incorporated with phenolic-rich extract of *M. comersonii* pulp prevented enzymatic browning in fresh-cut apples and *baroa* potatoes and improved the shelf life of the tested horticultural products. In addition, the edible coating was also able to reduce up to 2 log CFU/g of aerobic mesophiles of apples after 15 days of storage.

This study demonstrates the importance of research related to food preservation with edible coatings added with natural compounds. This work is a starting point towards future applications of apricot extract in the food industry.

## Figures and Tables

**Table 1:** Quantification and mass spectra of phenolic compounds identified in *M. comersonii* extract analyzed by LC-qTOF-MS/MS.

RT (min)	Molecular ion (m/z)	MS/MS (m/z)	Phenolic compound	Amount (*μ*g/mL)
2.6	343.1038 169.0324	191 125/85	3-O-galloyl quinic acid ^(1)^ Gallic acid^∗^^(2)^	90.73 ± 6.56^(3)^
8.9	479.1331	316/271	Myricetin-3-glucoside^∗^	7.05 ± 0.32
10.2	609.2073	301	Quercetin rutinoside^∗^	-
10.5	463.1361	301/271/163/151	Quercetin-3-glucoside^∗^	5.72 ± 0.22
11.9	447.1392	285/225	Kaempferol-3-glucoside^∗^	1.16 ± 0.04
15.3	625.1854	479/316	Myricetin glycoside (possible glycormanoside)	1.39 ± 0.10^(4)^

RT: retention time. ^∗^Identity confirmed using commercial standard; (1) J. Agric Food Chem. 2007, 55, 2797-2807; (2) https://massbank.eu/MassBank/RecordDisplay?id=PR308148 (accessed 15/01/2022); ^∗∗^(3) expressed as gallic acid; (4) expressed as myricetin-3-glucoside. -Trace amount of quercetin rutinoside, not quantifiable.

**Table 2:** Content of antioxidant activity of *M. comersonii* extracts.

Content of extracts	Pulp (M ± SD)	Seed (M ± SD)
Antioxidant activity—DPPH (EC50 g of fresh sample/g of DPPH)	478.62 ± 7.14^b^	1431.66 ± 7.14^a^
Antioxidant activity—ABTS (*μ*M of trolox/g of fresh sample)	77.89 ± 9.09^b^	248.78 ± 9.09^a^

M ± SD: means ± standard deviation. Means followed by different letters in the same line differ (*p* < 0.05) by Tukey's test. For more details on EC50, please refer to Material and Methods section.

**Table 3:** Microbial inhibition (inhibitory potential—IP) of *M. comersonii* extracts.

Bacteria	Phenolic-rich extract					
	Pulp			Seed		
	Concentration (mg GAE/g of pulp)	Extract dilution	IP	Concentration (mg GAE/g of pulp)	Extract dilution	IP
*E. coli* ATCC 25922	8.8	1 : 2	6.78	3.29	1 : 2	2.45
	4.4	1 : 4	5.65	1.65	1 : 4	0.87
	2.2	1 : 8	4.52	0.82	1 : 8	0.42
	1.1	1 : 16	1.50	0.41	1 : 16	0.15

*L. monocytogenes* ATCC 35152	8.8	1 : 2	1.60	3.29	1 : 2	3.20
	4.4	1 : 4	N.I	1.65	1 : 4	1.78
	2.2	1 : 8	N.I	0.82	1 : 8	1.28
	1.1	1 : 16	N.I	0.41	1 : 16	0.48

*P. aeruginosa* PA14	8.8	1 : 2	2.30	3.29	1 : 2	1.93
	4.4	1 : 4	1.11	1.65	1 : 4	1.40
	2.2	1 : 8	0.86	0.82	1 : 8	0.58
	1.1	1 : 16	0.58	0.41	1 : 16	0.24

*S.* Typhimurium ATCC 14028	8.8	1 : 2	2.49	3.29	1 : 2	2.20
	4.4	1 : 4	1.77	1.65	1 : 4	1.91
	2.2	1 : 8	1.46	0.82	1 : 8	0.67
	1.1	1 : 16	0,25	0.41	1 : 16	0,52

*S. aureus* ATCC 6538P	8.8	1 : 2	6.55	3.29	1 : 2	7.00
	4.4	1 : 4	6.45	1.65	1 : 4	6.88
	2.2	1 : 8	5.97	0.82	1 : 8	2.38
	1.1	1 : 16	2.74	0.41	1 : 16	0.59

IP was calculated as in Santos et al. [16] as follows: “IP equal to 1 indicates 10-fold inhibition compared to the control, due to logarithmic scale. N.I.: no inhibition. ^∗^We considered no inhibition when bacterial populations between treatment and control differed by less than twofold (IP < 0.301)”.

**Table 4:** Microbial inhibition, as determined by the inhibitory potential (IP), of the edible coatings incorporated with pulp or seed phenolic extracts of *M. comersonii* against selected microorganisms.

Bacteria	Edible coating with phenolic-rich extract			
	Pulp		Seed	
	Concentration (%)	IP	Concentration (%)	IP
*E. coli* ATCC 25922	9	2.19	9	2.25
*L. monocytogenes* ATCC 35152	9	2.00	9	2.25
*S.* Typhimurium ATCC 14028	9	2.01	9	2.28
*S. aureus* ATCC 6538P	9	2.31	9	2.35
*P. aeruginosa* PA14	9	2.21	9	2.25

An IP of 1 denotes a 10-fold inhibition.

**Table 5:** Browning index of minimally processed apples and *baroa* potatoes incorporated with edible coatings.

Food matrix	Treatment	Time (days)				
		3	6	9	12	15
Apple	AC	1.44^Ae^	4.76^Ad^	8.62^Ac^	12.13^Ab^	17.30^Aa^
	AE	1.39^Ad^	4.28^Ac^	6.54^Abc^	8.89^Bb^	12.22^Ba^
*Baroa* potato	PC	4.45^Ae^	8.52^Ad^	14.67^Ac^	20.77^Ab^	23.04^Aa^
	PE	2.82^Be^	7.73^Bd^	11.70^Bc^	15.71^Bb^	20.09^Ba^

AC: apple control treatment without the addition of phenolic-rich extract. AE: apple edible treatment with edible coatings incorporated with phenolic-rich extracts of *M. comersonii* pulp. PC: potato control treatment without the addition of phenolic-rich extract. PE: potato edible treatment with edible coatings incorporated with phenolic-rich extracts of *M. comersonii* pulp. Different capital letters differ (*p* < 0.05) in the same column, for the same food matrix. Different lowercase letters differ (*p* < 0.05) on the same line, for the same food matrix.

**Table 6:** Aerobic mesophilic count (log CFU/g) in minimally processed apples and *baroa* potatoes incorporated with edible coatings during 15 days of storage.

Food matrix	Treatment	Time (days)					
		0	3	6	9	12	15
Apple	AC	2.89^A^	3.18^A^	4.62^A^	5.23^A^	6.73^A^	7.95^A^
	AE	2.05^A^	2.84^A^	3.17^A^	4.15^A^	4.95^B^	5.54^B^
Baroa potato	PC	3.82^A^	5.01^A^	5.94^A^	7.37^A^	7.97^A^	8.56^A^
	PE	4.18^A^	4.49^A^	5.97^A^	8.03^A^	8.45^A^	9.03^A^

AC: apple control treatment without addition of phenolic-rich extract. AE: apple edible treatment with edible coatings incorporated with phenolic-rich extracts of *M. comersonii* pulp. PC: potato control treatment without addition of phenolic-rich extract. PE: potato edible treatment with edible coatings incorporated with phenolic-rich extracts of *M. comersonii* pulp. Different capital letters differ (*p* < 0.05) in the same column, for the same food matrix.

## Data Availability

The data used to support the findings of this study are available to interested readers upon reasonable request.

## Supplementary Materials

Supplementary Materials Supplementary Figures show results of LC-qTOF-MS/MS that detail the mass spectrum obtained in the study.
