# Supplementary material for: Antimicrobial and Antioxidant Activity of Apricot (Mimusopsis comersonii) Phenolic-Rich Extract and Its Application as an Edible Coating for Fresh-Cut Vegetable Preservation
Source: Biomed Res Int. 2022 Oct 21;2022:8440304. doi: 10.1155/2022/8440304 (PMC9616676; doi:10.1155/2022/8440304)
Supplement: Supplementary Materials — Supplementary Figures show results of LC-qTOF-MS/MS that detail the mass spectrum obtained in the study. [file 8440304.f1.docx]

**Supplementary material**

**A**

**B**

**C**

**D**

**E**

**F**

**G**

**Supplementary Figure 1**: Mass spectrum obtained by LC-qTOF-MS/MS of pulp phenolic extract of *M. comersonii*. Peaks identified: (A) 3-O-galloyl quinic acid; (B) Gallic acid; (C) myricetin-3-glucoside; (D) quercetin rutinoside; (E) quercetin-3-glucoside; (F) kaenpferol-3-glucoside; (G) myricetin glycoside.
